# Cryoablation of the Infrapatellar Branch of the Saphenous Nerve Identified by Non-Invasive Peripheral Nerve Stimulator for the Treatment of Non-Surgical Anterior Knee Pain: A Case Series and Review of the Literature

**DOI:** 10.7759/cureus.8747

**Published:** 2020-06-21

**Authors:** Brian C McLean, Conner D Nguyen, David P Newman

**Affiliations:** 1 Anesthesiology, Interdisciplinary Pain Management Center, Tripler Army Medical Center, Honolulu, USA; 2 Pain Management, Interdisciplinary Pain Management Center, Tripler Army Medical Center, Honolulu, USA; 3 Pain Management-Physiotherapy, Interdisciplinary Pain Management Center, Tripler Army Medical Center, Honolulu, USA

**Keywords:** cryoablation, knee pain, saphenous neuralgia, minimally invasive, interventional pain, rehabilitation

## Abstract

Chronic, non-surgical, non-specific anterior knee pain is a common source of functionally limiting chronic ailment, especially in a young athletic and active-duty military population. The infrapatellar branch of the saphenous is becoming a common therapeutic target for the diagnosis and treatment of anterior knee pain. It is a nerve commonly injured during knee surgeries and trauma, resulting in neuroma formation and chronic neuropathic pain states, and it can also transmit nociceptive input from patients with non-surgical anterior knee pain of multiple etiologies. Several methods have been employed to treat this condition. After the diagnosis of infrapatellar saphenous neuralgia, the nerve is safely ablated using radiofrequency ablation, neurolytic solutions, and, most recently, cryoablation using the handheld iovera® cryoablation system (Myoscience, Inc. Fremont, CA). Cryoablation is an attractive technique because it is minimally invasive, not permanent, and well tolerated by the patient with only local anesthesia. We have previously described a technique using a non-invasive peripheral nerve stimulator to identify and treat the exact location of the nerve more precisely, thereby optimizing treatment success and procedural simplicity. This case series illustrates our initial use and success with this technique. Further follow-up and randomized sham-controlled trials are also planned.

## Introduction

Anterior knee pain is a common clinical condition with a prevalence ranging from 11 to 22.7% in the general population and 28.9% among adolescents [1,2]. A high incidence of anterior knee pain is seen especially in elite athletes, military recruits, and women [3-5]. Anterior knee pain is considered idiopathic due to often vague complaints of retropatellar or peripatellar pain that develop insidiously due to multiple potential causes (i.e., overuse, mechanical, inflammatory, degenerative, and neoplastic) [6].

Diagnostic workup for anterior knee pain can be challenging given the lack of consensus regarding the specific pathogenesis [7] and the possibility of causative factors being due to a biomechanical fault in other anatomical locations [8]. The utility of imaging in non-acute knee pain is limited if intra-articular or extra-articular pathology is not present [9]. Plain-film X-rays are highly sensitive in cases of acute knee pain when using the Ottawa knee rules. MRI has demonstrated high sensitivity for meniscal and ligamentous injuries [10]; however, in older patients, MRI has shown no structural damage in 22% of those who complained of anterior knee pain [11].

A structured clinical examination matched with the patients’ symptoms may assist in identifying structural and biomechanical faults in the lower extremity. However, reliance on common clinical tests performed during the objective examination may only be helpful in ruling out other similar conditions [12]. It may be more appropriate to consider anterior knee pain as a diagnosis of exclusion.

Multimodal physical therapy has been shown to be effective in the management of anterior knee pain [13]. Several high-quality studies support six to eight weeks of physical therapy exercise to the structures surrounding the knee and/or hip [14]. Passive treatments include patellar taping, orthoses, foot orthotics, and acupuncture. Despite promising results from active and passive treatments, more than 50% of patients report persistent pain several years after the onset [15].

Many techniques have been suggested and trialed for patients for whom rest, conservative care, physical therapy, and rehabilitative efforts have failed. These include oral medications, bracing, acupuncture, transcutaneous electrical nerve stimulation (TENS) therapy, massage, corticosteroid injections, viscous-supplementation, prolotherapy, platelet-rich plasma therapy, and nerve ablation procedures [16-18]. There is confounding evidence in the literature regarding the short- and long-term benefits from each of these regenerative and palliative procedures. We feel that in our relatively young and active patient population, regenerative medicine is the desire of both patients and physicians; however, the data has not yet provided definitive evidence relating to this, and many patients still fail regenerative medicine procedures. In patients with chronic non-surgical anterior knee pain who have failed physical therapy and other conservative modalities of pain management listed above, we consider palliative neuroablative techniques as the next option. Given the simplicity of the procedure, patient acceptance, and our significant positive results as well as the absence of complications at our center or in the literature to date, we have been offering a prognostic infrapatellar branch of the saphenous nerve (IPBSN) block followed by cryoablation of this branch with the iovera® device (Myoscience, Inc. Fremont, CA). This procedure has been demonstrated to be effective in patients with symptoms of knee arthritis [18], and we have also found it effective for patients with chronic, non-specific, non-surgical anterior knee pain, as it provides them pain relief, functional improvement, and increased tolerance for rehabilitation.

## Case presentation

**Figure 1:**
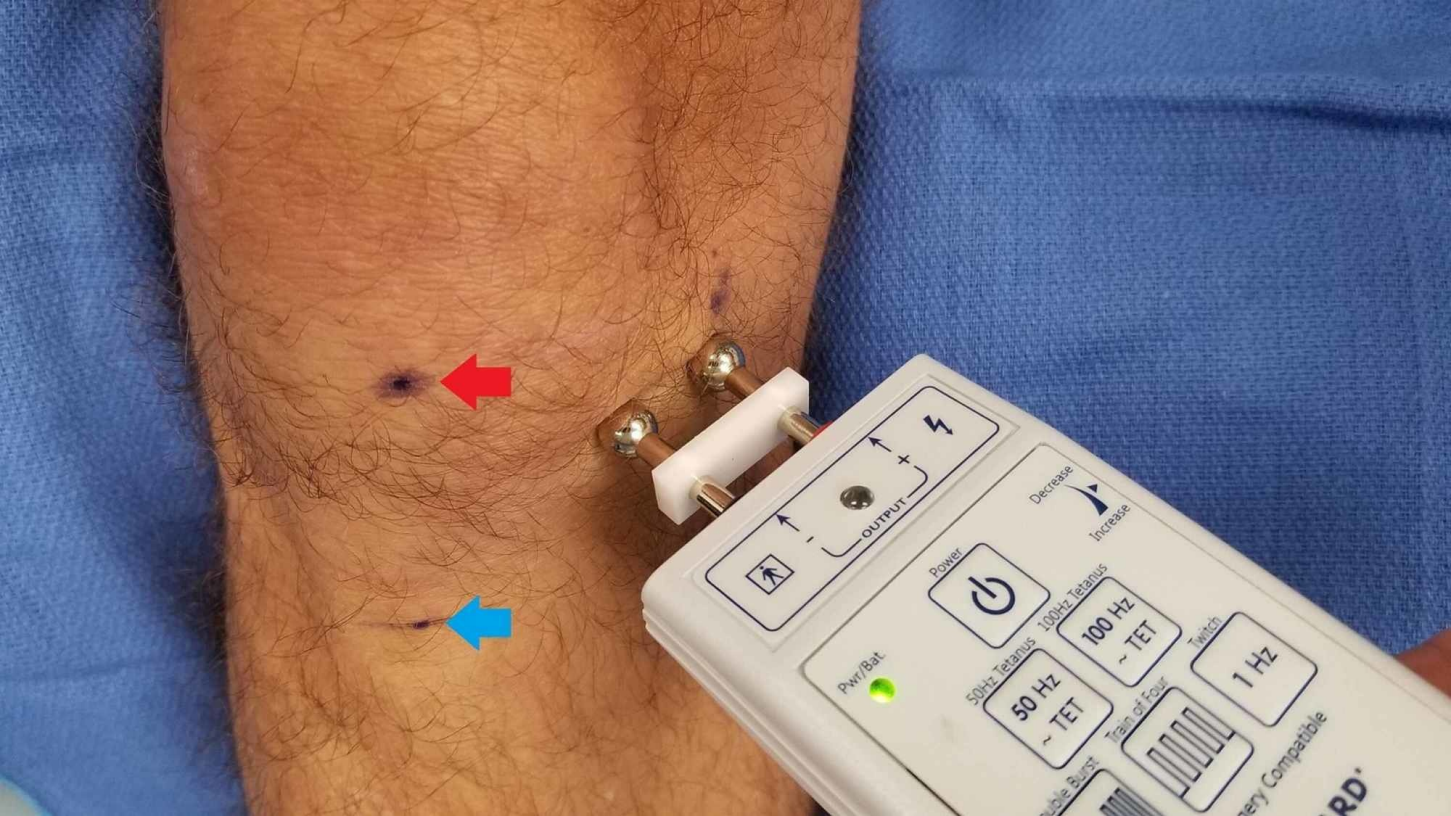
Scanning the knee to Identify the nerve Red arrow: the inferior pole of the patella. Blue arrow: tibial tubercle

**Figure 2:**
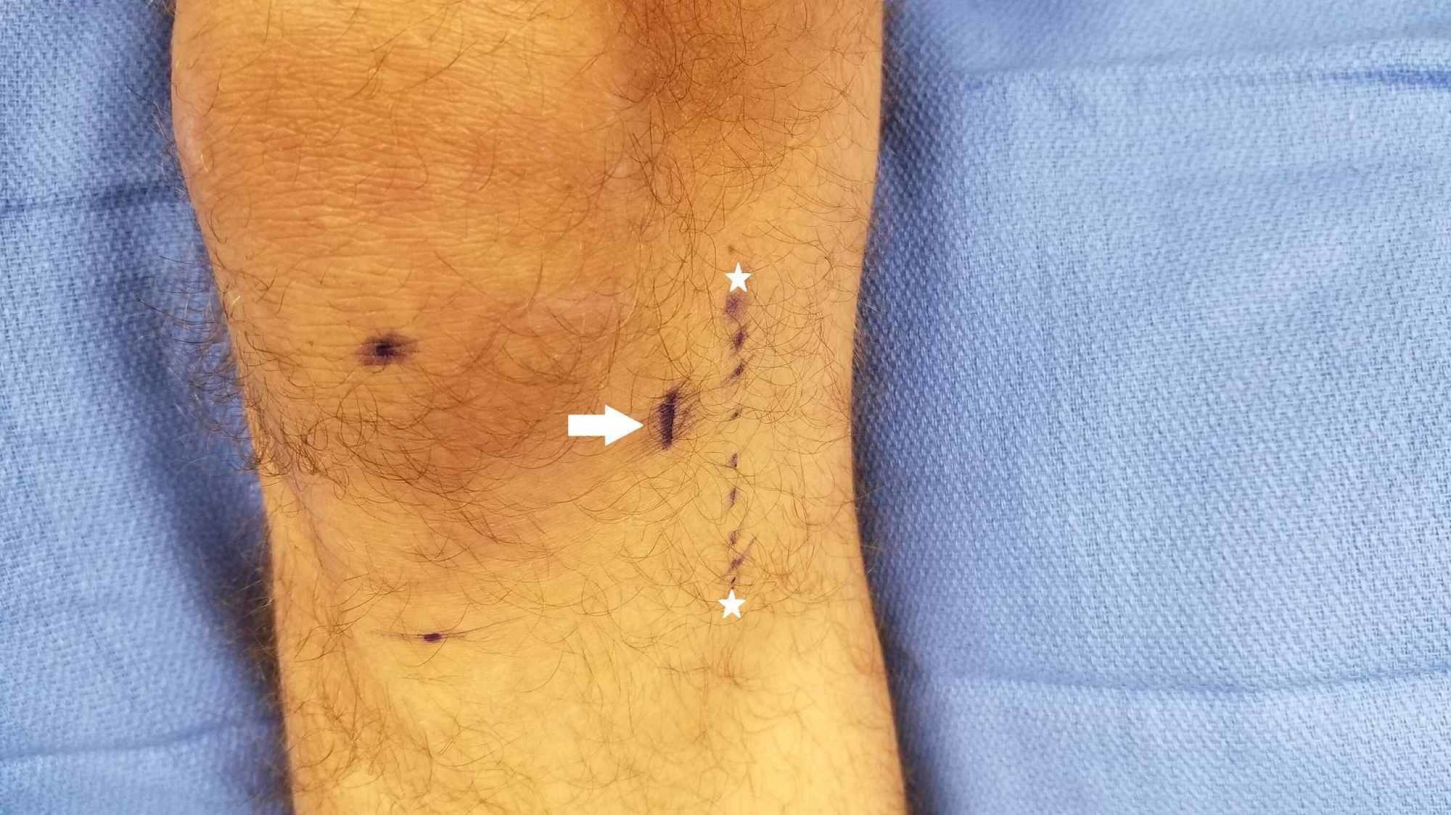
Identifying treatment line location White arrow: treatment line identified with a nerve stimulator. White stars: originally described treatment line

**Figure 3:**
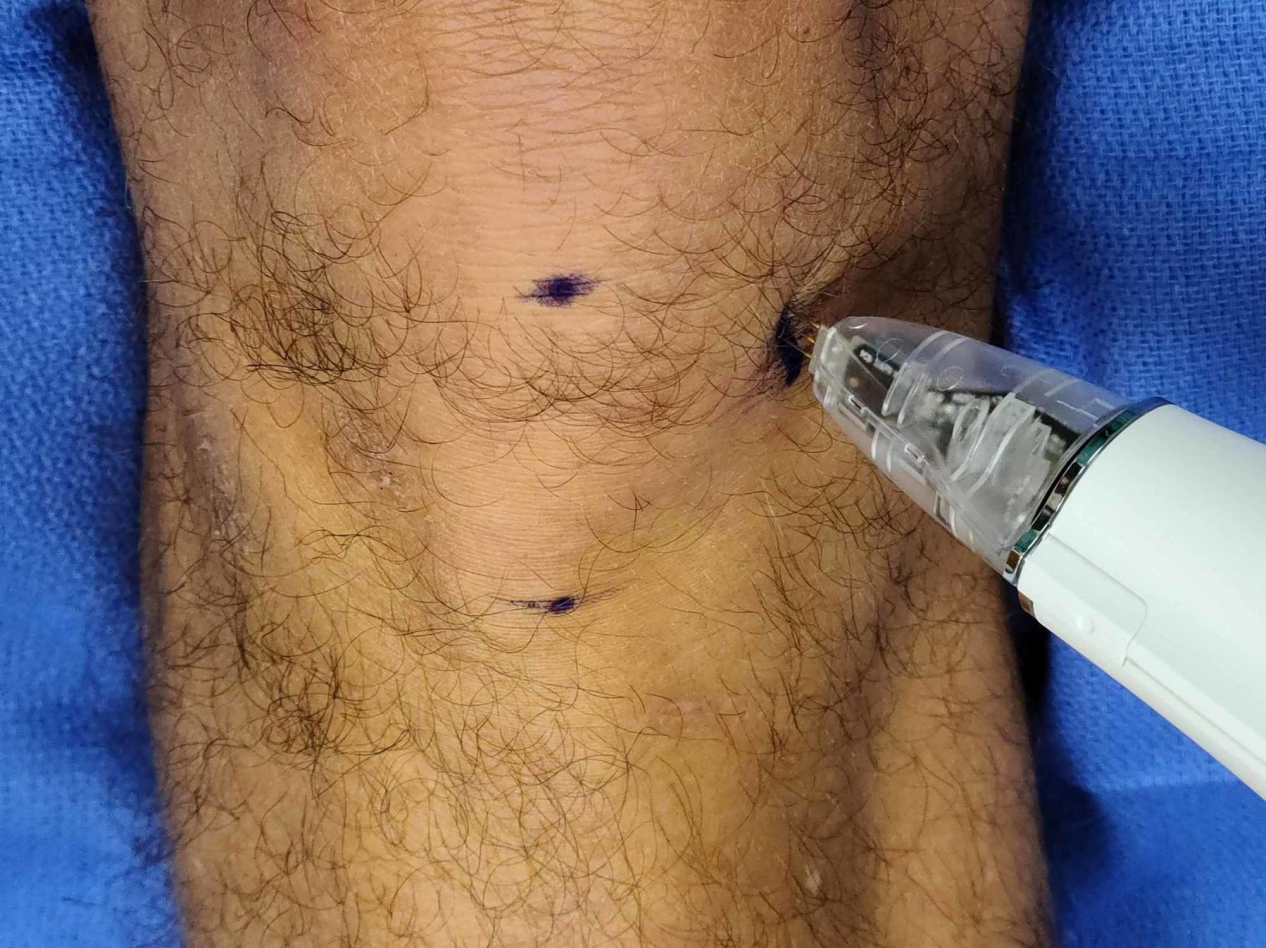
Treatment with iovera® device

After discussing the procedure with the patient and obtaining informed consent, a line is drawn 5 cm medial from the inferior pole of the patella and tibial tubercle. Next, the peripheral nerve stimulator with a transcutaneous bipolar probe is placed on the skin with a stimulus amplitude dial set approximately at 2, and then the 50-Hz tetanus setting is used to stimulate the nerve. The patient is instructed to indicate when they feel a pinch or tingling sensation. The device is used to scan up and down a vertical line approximately 5 cm from the patella and tibial tuberosity. If the patient does not feel the stimulation at this setting, the intensity is gradually increased in approximately 0.5 increments until the patient detects a sensation at one of the terminals. We have found that for our patient population, the stimulation threshold has been at approximately 3-4 on the dial. After the perception of the stimulus, we make a slow and deliberate scan (Figure 1) of the area medial to the patella and instruct the patient to tell you when they feel stimulation travel to the center of their knee or pain location as opposed to local stimulation. When this happens, we mark the location with a skin marker. Next, we confirm the location by stimulating at 0.5 cm above and below this mark to confirm the precise location of the point of maximal stimulation. This point should be the treatment location of the nerve. After marking this location (Figure 2), we use a 30-ga 1-inch needle to then localize this point with 1 ml of 2% lidocaine or 0.5% ropivacaine for the diagnostic block and then provide them with a pain diary and instructions to perform activities that would normally exacerbate their knee pain. If they have at least 50% improvement in pain and function while performing normally painful activities, we will offer them a trial of cryoablation. For the therapeutic treatment, the same exact technique is used as above, and 1 ml of 2% lidocaine is again utilized over the skin marking and cryoablation is performed using the iovera® Smart Tip (Figure 3), treating for 4-6 cycles with overlapping placements.

Table 1 lists the details of the last 22 patients that we treated in this manner after a positive prognostic block with greater than 50% pain relief for the duration of the local anesthetic. In the patients with a listed duration of relief, their data represents a repeat treatment and the duration of relief they had from their initial treatment. The remaining patients have not yet followed up to determine the duration of relief, but based on our experience of treating other patients over the past few years, the treatment duration can be safely assumed to be 3-12 months.

**Table 1:** Case series PFPS: patellofemoral pain syndrome; DVPRS: Defense and Veterans Pain Rating Scale

Case	Age, years	Diagnosis	Pre-procedure DVPRS score	Post-procedure DVPRS score	Duration of relief
1	47	PFPS chondromalacia	8	0	6 months
2	45	PFPS	7	0	9 months
3	36	Chondromalacia	6	0	6 months
4	40	Chondromalacia	4	1	6 months
5	40	Chondromalacia	8	1	
6	42	Chondromalacia	8	1	
7	33	Chondromalacia	9	0	
8	52	Non-specific, normal image	5	0	
9	46	Chondromalacia	6	0	
10	46	PFPS	7	0	
11	27	Non-specific, normal image	6	0	
12	48	Non-specific, normal image	4	0	
13	34	PFPS	7	1	
14	54	Chondromalacia	6	1	
15	24	PFPS	4	0	
16	32	PFPS	4	0	
17	43	Chondromalacia	4	0	
18	44	Chondromalacia	4	0	
19	35	Chondromalacia	4	0	
20	67	chondromalacia	6	0	
21	26	PFPS	6	0	
22	32	Chondromalacia	7	2	
23	53	Chondromalacia	5	0	

## Discussion

Anterior knee pain is among the most common knee conditions encountered by primary care physicians, orthopedic surgeons, and sports medicine specialists. This case series served primarily as a feasibility study to demonstrate the relative simplicity and effectiveness of a novel IPBSN identification and treatment technique and investigate the therapeutic benefit of applying cryoablative treatment at this location in patients suffering from chronic, non-specific anterior knee pain. Given the significant anatomic variability of the IPBSN course [19], the authors employed a novel technique described previously [20] to locate the IPBSN and improve treatment outcomes more precisely. 

Twenty-three patients (each receiving unilateral knee treatment) who had a positive response to diagnostic IPBSN block received cryoablative treatment in this case series. A response was considered positive when there was an equal to or greater than 50% drop in Defense and Veterans Pain Rating Scale (DVPRS) pain score either at rest or during the patient’s painful activity. The patients’ age ranged from 24 to 67 with a mean age of 42.5 years. Thirteen patients had a diagnosis of chondromalacia patellae and six patients had a diagnosis of patellofemoral pain syndrome (PFPS). One patient was diagnosed with both chondromalacia patellae and PFPS. Two patients had normal-appearing imaging studies and their condition was designated as “other” non-specific anterior knee pain.

At the time of this case series submission, four patients had returned for repeat cryoablation of the same IPBSN after regeneration of the target nerve and return of their pain. Their duration of relief (>50% DVPRS drop from baseline) ranged from six to nine months. The same procedure was repeated for these patients with immediate post-procedure pain relief. The remaining 19 patients have not yet returned for follow-up or repeat treatment after one to nine months of initial treatment, possibly indicating that the pain has not returned to pre-treatment levels.

This case series has some limitations, including those that are inherent to case series in general, such as the lack of control subjects making case series prone to selection bias. In addition, the lack of follow-up makes it difficult to accurately determine the actual procedure success and delayed complication rate. Additionally, this case series lacked uniformed pre-defined follow-up duration after treatment because our patients have generally tended to appear for follow-up or repeat treatment only when they feel their symptoms have returned to a “significant” level. Future studies should have pre-determined interval follow-ups to quantify the loss to follow-up, which can be an important factor in assessing study quality. In addition, pre-set follow-ups may identify cases where patients no longer experience satisfactory pain relief but still failed to return for re-evaluation or repeat treatment for whatever reason.

This case series had one sole outcome measure: change in DVPRS numerical score. Future study endpoints should include functional measures such as the Western Ontario and McMaster Universities Osteoarthritis Index (WOMAC) as improvement in function in conjunction with pain relief has been widely accepted to yield a more meaningful assessment of the quality of life than pain scores alone.

Moreover, future studies will ideally be randomized, double-blind, and sham-controlled in design. These studies could include the following cohorts: 1. cryoablation utilizing the novel nerve identification technique; 2. cryoablation using the manufacturer’s (iovera®) recommended treatment location; 3. sham ablation utilizing the novel nerve identification technique; and 4. sham ablation using the iovera®-recommended treatment location.

## Conclusions

While acknowledging its limitations, we believe this case series demonstrates positive outcomes of a novel diagnostic and therapeutic procedure as part of a high-impact treatment in patients who had an inadequate response to rehabilitation and conservative treatment and limited alternative options for pain relief and functional improvement. We hope that this case series and procedure report will encourage physicians/researchers to conduct more advanced studies, which would lead to a widespread acceptance of this quick, seemingly effective, and well-tolerated technique.
